# Sex Differences in Diabetes Prevalence, Comorbidities, and Health Care Utilization among American Indians Living in the Northern Plains

**DOI:** 10.1093/cdn/nzz089

**Published:** 2019-08-06

**Authors:** Kimberly R Huyser, Jennifer Rockell, Valarie Blue Bird Jernigan, Tori Taniguchi, Charlton Wilson, Spero M Manson, Joan O'Connell

**Affiliations:** 1 Department of Sociology, University of New Mexico, Albuquerque, NM, USA; 2 Telligen, Greenwood Village, CO, USA; 3 Center for Indigenous Health Research and Policy, Oklahoma State University Center for Health Sciences, Tulsa, OK, USA; 4 Indian Health Service, Phoenix, AZ, USA; 5 Centers for American Indian and Alaska Native Health, Colorado School of Public Health, University of Colorado Anschutz Medical Campus, Aurora, CO, USA

**Keywords:** diet-related disparities, cardiovascular disease, diabetes mellitus, health services, American Indians, health care costs

## Abstract

**Background:**

The American Indian (AI) population experiences significant diet-related health disparities including diabetes and cardiovascular disease (CVD). Owing to the relatively small sample size of AIs, the population is rarely included in large national surveys such as the NHANES. This exclusion hinders efforts to characterize potentially important differences between AI men and women, track the costs of these disparities, and effectively treat and prevent these conditions.

**Objective:**

We examined the sex differences in diabetes prevalence, comorbidity experience, health care utilization, and treatment costs among AIs within a Northern Plains Indian Health Service (IHS) service unit.

**Methods:**

We assessed data from a sample of 11,144 persons using an IHS service unit in the Northern Plains region of the United States. Detailed analyses were conducted for adults (*n* = 7299) on prevalence of diabetes by age and sex. We described sex differences in comorbidities, health care utilization, and treatment costs among the adults with diabetes.

**Results:**

In our sample, adult men and women had a similar prevalence of diabetes (10.0% and 11.0%, respectively). The prevalence of CVD among men and women with diabetes was 45.7% and 34.0%, respectively. Among adults with diabetes, men had a statistically higher prevalence of hypertension and substance use disorders than women. The men were statistically less likely to have a non–substance use mental health disorder. Although men had higher utilization and costs for hospital inpatient services than women, the differences were not statistically significant.

**Conclusions:**

In this AI population, there were differences in comorbidity profiles between adult men and women with diabetes, which have differential mortality and cost consequences. Appropriate diabetes management addressing gender-specific comorbidities, such as substance use disorders for men and non–substance use mental health disorders for women, may help reduce additional comorbidities or complications to diabetes.

## Introduction

American Indian and Alaska Native peoples (AI/ANs) are more than twice as likely to experience diet-related health disparities [e.g., diabetes, obesity, or cardiovascular disease (CVD)] as the general US population (1, 2). From 1990 to 2009, AI/ANs experienced 46% higher mortality per 100,000 people than non-Hispanic whites, due in part to disparities in mortality associated with diabetes, stroke, and CVD (3). AI/ANs with diabetes are 3–4 times more likely to experience mortality than their white peers (4). AI/ANs report the highest prevalence of CVD among US population groups, and CVD is also a leading cause of death (5–7). Sex differences in mortality associated with diabetes and CVD have been observed in the general population and among AI/ANs (8, 9).

Although there are documented sex differences in health status and health care utilization patterns in the US general population, the literature on sex differences among AI/ANs is still developing (10, 11). The Strong Heart Study (SHS) has found that AI men and women had similar blood pressure levels and that elderly women experienced higher concentrations of cholesterol and triglycerides than men (12). Within the SHS, although men had absolute rates of CVD that were higher than women’s, diabetes conferred a greater risk of CVD among AI women than men (13). From 1990 to 2009, AI men had a higher diabetes-related mortality rate than AI women (14). These sex difference trends extend to health care service use where females with diabetes use more health care services than males with diabetes (15).

To contribute to this understanding, we examine the sex differences in diabetes prevalence, comorbidity experience, health care utilization, and treatment costs within a Northern Plains Indian Health Service (IHS) service area—the IHS is an agency within the Department of Health and Human Services and the principal federal health care provider for AI/ANs (16–18).

## Methods

### Data sources and approvals

The data for the Northern Plains IHS Service Unit for fiscal year 2010 (FY2010) come from the IHS Improving Healthcare Delivery Data Project (Data Project), which was created to provide a longitudinal data infrastructure of sufficient size and scope that it may be used for program planning and evaluation with a specific focus on chronic diseases. The data infrastructure includes 4 existing types of IHS electronic data: *1*) the National Patient Information Reporting System (NPIRS), which includes utilization data for IHS services; *2*) Purchased/Referred Care (PRC), which includes information on specialty inpatient and outpatient services obtained from non-IHS providers and paid for by the IHS; *3*) Centers for Medicare & Medicaid Services (CMS) Cost Reports (Cost Reports); and *4*) IHS procurement data for prescribed medication costs. The data used in this analysis are from Phase 1 of the Data Project and include FY2010 measures. The FY2010 Northern Plains service area population includes 11,144 persons.

The project is based at the Centers for American Indian and Alaska Native Health (CAIANH), Colorado School of Public Health, University of Colorado Anschutz Medical Campus. The protocol was reviewed by the IHS National Institutional Review Board (IRB), the Colorado Multiple IRB (i.e., university IRB), 1 regional IHS IRB, and 6 Tribal IRBs. Tribal Councils have provided approvals though resolutions and letters for project involvement. In addition, a data agreement was put in place with the IHS and 1 Tribal organization.

### Measures

#### Demographic characteristics

NPIRS demographic information includes age, gender, and geographic information.

#### Health status

We used Sightlines DxCG Risk Solutions software to identify diagnosed conditions a patient may have (19). This software is used by CMS and other health care organizations to understand the morbidity burden of a population; it classifies the international classification of diseases, ninth revision, clinical modification (ICD-9-CM) diagnostic codes recorded in the NPIRS and PRC service utilization data into condition categories. We assessed the prevalence of diabetes, CVD, and other conditions (e.g., hypertension, renal disease/failure, amputations, behavioral health disorders, liver disease) based on those categories.

#### Inpatient services

NPIRS and PRC inpatient service measures include the number of admissions and inpatient days.

#### Outpatient services

NPIRS includes >120 outpatient clinic codes. We used clinic and provider codes to categorize outpatient services into service categories to report IHS outpatient utilization of these. They included emergency, urgent, primary, specialty (e.g., cardiology, nephrology, orthopedic), dental, eye care (i.e., optometry, ophthalmology), foot care (i.e., podiatry, diabetes foot clinic), behavioral health, physical therapy and other rehabilitative services, public health nurse, and home services. Five of the service categories were designated as education, case management, and advanced practice pharmacy services (ECP). Education services include those for diabetes, nutrition, and other types of education. Outpatient services for gynecology, obstetrics, and women's health screening were excluded to facilitate comparisons by sex in outpatient service use. Procedure codes were used in addition to clinic and provider codes to identify ECP services.

#### For IHS-provided services

During Phase 1, we developed algorithms to use FY2010 Cost Report data, NPIRS utilization data for all persons who used services, and local data for the Service Unit to estimate site-specific costs for the provision of different types of IHS-provided services (e.g., 1 inpatient day; 1 emergency, urgent, primary, or specialty visit; 1 medication). The site-specific service cost estimates for each type of visit included costs associated with ancillary services (e.g., laboratory, radiology). The expert opinions of IHS/Tribal fiscal and medical personnel and CAIANH were used to guide allocations of some costs (e.g., ancillary costs, medical supplies) across service types when data were not available.

#### Total IHS treatment costs

Treatment costs for IHS-provided services for each person were estimated based on his/her utilization of inpatient and outpatient services (e.g., 1 urgent care visit and 4 primary care visits) and the estimated average costs of providing those services in the Service Unit where he/she lived. IHS-paid amounts for PRC services were used to estimate his/her costs of inpatient and outpatient services that occurred at non-IHS providers. Total IHS treatment costs for each person were calculated by summing his/her estimated costs for IHS- and non-IHS-provided services. It is important to note that IHS treatment costs do not include costs for services obtained at non-IHS providers and not paid for by IHS. For example, the costs exclude the majority of costs associated with renal dialysis.

We used SAS statistical software version 9.4 (SAS Institute, Inc.) (20) to conduct the analyses to describe the health status, service utilization, and treatment costs for the AI adults living in the Northern Plains IHS Service Unit. We evaluated the statistical differences between men and women using CIs calculated at the 95% level, with nonoverlapping CIs indicating statistical significance (21). We report on outpatient service utilization for selected services. Utilization information for services not reported may be obtained directly from the authors.

## Results

Among all persons, the prevalence of diabetes was 10.5%. The overall prevalence of diabetes did not differ by gender, nor were sex differences found for any age group (**Table 1**). Owing to the small number of youth with diabetes, we examined comorbidities, health care utilization, and IHS treatment costs only for adults with diabetes.

**TABLE 1:** Prevalence of diabetes by age and gender among American Indians living in a Northern Plains Service Unit, fiscal year 2010

	Total persons	Persons with diabetes			Total males	Males with diabetes			Total females	Females with diabetes		
Age group, y	*n*	*n*	Percentage	95% CI	*n*	*n*	Percentage	95% CI	*n*	*n*	Percentage	95% CI
0–17	3845	9	0.2	(0.1, 0.4)	1955	5	0.3	(0.0, 0.5)	1890	4	0.2	(0.0, 0.4)
18–34	3097	120	3.9	(3.2, 4.6)	1559	55	3.5	(2.6, 4.4)	1538	65	4.2	(3.2, 5.2)
35–44	1129	162	14.3	(12.3, 16.4)	552	80	14.5	(11.5, 17.4)	577	82	14.2	(11.4, 17.1)
45–54	1435	272	19.0	(16.9, 21.0)	684	131	19.2	(16.2, 22.1)	751	141	18.8	(16.0, 21.6)
55–64	851	255	30.0	(26.9, 33.0)	379	108	28.5	(23.9, 33.1)	472	147	31.1	(27.0, 35.3)
≥65	787	354	45.0	(41.5, 48.5)	362	169	46.7	(41.5, 51.8)	425	185	43.5	(38.8, 48.3)
All ages	11,144	1172	10.5	(9.9, 11.1)	5491	548	10.0	(9.2, 10.8)	5653	624	11.0	(10.2, 11.9)
All ages with age adjustment											9.8	(9.0, 10.6)

Among the AI adults with diabetes, the most common comorbidities experienced by our sample overall were hypertension (75.4%), followed by CVD (39.5%), non–substance use mental health disorders (25.3%), and tobacco use disorders (21.1%) (**Table 2**). Prevalence of comorbidities by sex suggests differing experiences of diabetes and its complications. Among all ages, AI men experienced a statically higher prevalence of hypertension, CVD, and substance use disorders than AI women. AI women experienced a statistically higher prevalence of non–substance use mental health disorders relative to AI men. Among ages 55 y and older, we saw similar trends. AI men who were ages 55 y and older had higher levels of hypertension, CVD, and substance use disorders than AI women ages 55 y and older. For women ages 55 y and older, non–substance use mental health disorders were more common than among men of the same age.

**TABLE 2:** Prevalence of comorbidities among American Indian adults with diabetes by age and gender, Northern Plains Service Unit, fiscal year 2010

	All adults			Men			Women			
Health condition	*n*	Percentage	95% CI	*n*	Percentage	95% CI	*n*	Percentage	95% CI	Statistically significant
All ages										
Hypertension	877	75.4	(72.9, 77.9)	449	82.7	(79.5, 85.9)	428	69.0	(65.4, 72.7)	*
Cardiovascular conditions^1^	459	39.5	(36.7, 42.3)	248	45.7	(41.5, 49.9)	211	34.0	(30.3, 37.8)	*
Renal disease/failure	186	16.0	(13.9, 18.1)	101	18.6	(15.3, 21.9)	85	13.7	(11.0, 16.4)	
ESRD^2^	54	4.6	(3.4, 5.9)	26	4.8	(3.0, 6.6)	28	4.5	(2.9, 6.2)	
Neuropathy	339	29.1	(26.5, 31.8)	166	30.6	(26.7, 34.5)	173	27.9	(24.4, 31.4)	
Amputations^3^	31	2.7	(1.7, 3.6)	22	4.1	(2.4, 5.7)	9	1.5	(0.5, 2.4)	
Transplants^4^	11	0.9	(0.4, 1.5)	6	1.1	(0.2, 2.0)	5	0.8	(0.1, 1.5)	
Non–substance use mental health disorders	294	25.3	(22.8, 27.8)	106	19.5	(16.2, 22.9)	188	30.3	(26.7, 34.0)	*
Depression^5^	196	16.9	(14.7, 19.0)	66	12.2	(9.4, 14.9)	130	21.0	(17.8, 24.2)	*
Substance use disorders	81	7.0	(5.5, 8.4)	53	9.8	(7.3, 12.3)	28	4.5	(2.9, 6.2)	*
Tobacco use disorder	245	21.1	(18.7, 23.4)	107	19.7	(16.3, 23.1)	138	22.3	(19.0, 25.5)	
Liver disease	104	8.9	(7.3, 10.6)	57	10.5	(7.9, 13.1)	47	7.6	(5.5, 9.7)	
Age < 55 y										
Hypertension	348	62.8	(58.8, 66.9)	196	73.7	(68.4, 79.0)	152	52.8	(47.0, 58.6)	
Cardiovascular conditions^1^	104	18.8	(15.5, 22.0)	57	21.4	(16.5, 26.4)	47	16.3	(12.0, 20.6)	
Renal disease/failure	39	7.0	(4.9, 9.2)	28	10.5	(6.8, 14.2)	11	3.8	(1.6, 6.0)	
ESRD	11	2.0	(0.8, 3.2)	7	2.6	(0.7, 4.6)	4	1.4	(0.0, 2.7)	
Neuropathy	113	20.4	(17.0, 23.8)	60	22.6	(17.5, 27.6)	53	18.4	(13.9, 22.9)	
Amputations^3^	9	1.6	(0.6, 2.7)	6	2.3	(0.5, 4.1)	3	1.0	(−0.1, 2.2)	
Transplants^4^	2	0.4	(−0.1, 0.9)	1	0.4	(−0.4, 1.1)	1	0.3	(−0.3, 1.0)	
Non–substance use mental health disorders	129	23.3	(19.8, 26.8)	46	17.3	(12.7, 21.9)	83	28.8	(23.6, 34.1)	
Depression^5^	85	15.3	(12.3, 18.4)	25	9.4	(5.9, 12.9)	60	20.8	(16.1, 25.6)	
Substance use disorders	62	11.2	(8.6, 13.8)	38	14.3	(10.1, 18.5)	24	8.3	(5.1, 11.5)	
Tobacco use disorder	140	25.3	(21.6, 28.9)	63	23.7	(18.5, 28.8)	77	26.7	(21.6, 31.9)	
Liver disease	55	9.9	(7.4, 12.4)	27	10.2	(6.5, 13.8)	28	9.7	(6.3, 13.2)	
Age ≥55 y										
Hypertension	529	86.9	(84.2, 89.6)	253	91.3	(88.0, 94.7)	276	83.1	(79.1, 87.2)	*
Cardiovascular conditions^1^	355	58.3	(54.4, 62.2)	191	69.0	(63.5, 74.4)	164	49.4	(44.0, 54.8)	*
Renal disease/failure	147	24.1	(20.7, 27.5)	73	26.4	(21.1, 31.6)	74	22.3	(17.8, 26.8)	
ESRD	43	7.1	(5.0, 9.1)	19	6.9	(3.9, 9.9)	24	7.2	(4.4, 10.0)	
Neuropathy	226	37.1	(33.3, 41.0)	106	38.3	(32.5, 44.0)	120	36.1	(31.0, 41.3)	
Amputations^3^	22	3.6	(2.1, 5.1)	16	5.8	(3.0, 8.5)	6	1.8	(0.4, 3.2)	
Transplants^4^	9	1.5	(0.5, 2.4)	5	1.8	(0.2, 3.4)	4	1.2	(0.0, 2.4)	
Non–substance use mental health disorders	165	27.1	(23.6, 30.6)	60	21.7	(16.8, 26.5)	105	31.6	(26.6, 36.7)	*
Depression^5^	111	18.2	(15.2, 21.3)	41	14.8	(10.6, 19.0)	70	21.1	(16.7, 25.5)	
Substance use disorders	19	3.1	(1.7, 4.5)	15	5.4	(2.7, 8.1)	4	1.2	(0.0, 2.4)	*
Tobacco use disorder	105	17.2	(14.2, 20.2)	44	15.9	(11.6, 20.2)	61	18.4	(14.2, 22.6)	
Liver disease	49	8.0	(5.9, 10.2)	30	10.8	(7.1, 14.5)	19	5.7	(3.2, 8.2)	

^1^Cardiovascular conditions include ischemic heart disease, congestive heart failure and other forms of heart disease, cerebrovascular disease, and vascular disease.

^2^End-stage renal disease.

^3^The prevalence of amputations represents amputations noted on the utilization records during the specific fiscal years; providers may not have documented in the utilization record that a person had an amputation during previous years.

^4^Transplants include liver, heart, bone marrow, and others.

^5^Depression is one of the non-substance use mental health disorders included in the category “Mental health disorders”; other types of mental health disorders include anxiety, bipolar, and posttraumatic stress disorders.

We next examined health care utilization and IHS treatment costs. AI adults with diabetes averaged 2.5 d in the hospital during the year (**Table 3**). Although men had a higher mean number of inpatient days than women (2.9 compared with 2.2) and women had a slightly higher number of primary care visits than men (3.6 compared with 3.3), these differences were not statistically significant. All other health care service utilization was similar between men and women.

**TABLE 3:** Health service utilization and estimated mean IHS treatment costs among adults with diabetes by gender, Northern Plains Service Unit, fiscal year 2010^1^

	All adults			Men			Women		
Health Service Utilization	Number of visits	Utilization rate	95% CI	Number of visits	Utilization rate	95% CI	Number of visits	Utilization rate	95% CI
Inpatient services (excluding obstetric care)^2^									
IHS and non-IHS admissions	603	0.5	(0.4, 0.6)	308	0.6	(0.5, 0.7)	295	0.5	(0.4, 0.6)
IHS admissions	344	0.3	(0.2, 0.3)	169	0.3	(0.2, 0.4)	175	0.3	(0.2, 0.4)
Non-IHS admissions	259	0.2	(0.2, 0.3)	139	0.3	(0.2, 0.3)	120	0.2	(0.1, 0.3)
IHS and non-IHS inpatient days	2952	2.5	(2.0, 3.0)	1565	2.9	(2.1, 3.7)	1387	2.2	(1.6, 2.8)
IHS inpatient days	1675	1.4	(1.1, 1.8)	870	1.6	(1.0, 2.2)	805	1.3	(0.9, 1.7)
Non-IHS inpatient days	1277	1.1	(0.8, 1.4)	695	1.3	(0.9, 1.7)	582	0.9	(0.5, 1.3)
IHS outpatient services									
Urgent care	1978	1.7	(1.6, 1.8)	812	1.5	(1.3, 1.7)	582	1.9	(1.7, 2.1)
Emergency	3754	3.2	(2.9, 3.5)	1634	3.0	(2.6, 3.4)	1166	3.4	(3.0, 3.9)
Primary care^3^	4007	3.4	(3.3, 3.6)	1785	3.3	(3.0, 3.6)	2120	3.6	(3.3, 3.8)
Education, case management, and advanced practice pharmacy	148	0.1	(0.1, 0.2)	67	0.1	(0.0, 0.2)	2222	0.1	(0.1, 0.2)
**Estimated IHS treatment costs**		**Mean cost per adult**	**95% CI**		**Mean cost per adult**	**95% CI**		**Mean cost per adult**	**95% CI**
IHS and non-IHS Inpatient services (excluding obstetric care)		3633	(2832, 4434)		4312	(2998, 5626)		3038	(2071, 4005)
IHS inpatient services		2135	(1597, 2672)		2375	(1441, 3308)		1924	(1333, 2516)
Non-IHS inpatient services		1498	(960, 2037)		1938	(1133, 2742)		1113	(388, 1838)
IHS and non-IHS outpatient services		5047	(4634, 5461)		4933	(4442, 5424)		5148	(4501, 5794)
IHS outpatient services		3953	(3779, 4127)		3781	(3516, 4045)		4104	(3874, 4334)
Urgent care		232	(214, 250)		204	(179, 230)		257	(231, 282)
Emergency		995	(911, 1080)		973	(838, 1109)		1015	(910, 1120)
Primary care^3^		1367	(1293, 1441)		1304	(1193, 1415)		1422	(1323, 1521)
Education, case management, and advanced practice pharmacy		21	(11, 30)		20	(1, 38)		22	(14, 29)
Non-IHS outpatient services		1094	(737, 1452)		1152	(793, 1512)		1043	(450, 1637)
IHS treatment costs for IHS and non-IHS services^4^		10,825	(9814, 11,837)		11,277	(9693, 12,860)		10,430	(9132, 11,728)

^1^IHS, Indian Health Service; IT, IHS or Tribal facilities; PRC, Purchased Referred Care.

^2^Obstetric admissions were excluded from the IHS/Tribal admissions, they were not excluded from the PRC admissions.

^3^Primary care visits include diabetes clinic, preventive, and general office visits, and exclude obstetric, gynecological, and women's health screening visits.

^4^Costs for all IT and PRC services include inpatient, outpatient, and prescriptions dispensed at the pharmacy.

The FY2010 estimated IHS mean treatment cost per person for adults with diabetes was ${\$}$10,825: on average, ${\$}$3633 for inpatient services and ${\$}$5047 for outpatient services. Approximately 33.6% of total treatment costs were for inpatient service use.

Mean treatment costs for AI men and women were ${\$}$11,277 and ${\$}$10,430, respectively. Although total treatment costs differed by approximately ${\$}$900, this difference was not statistically significant. Mean outpatient treatment costs for men and women with diabetes were more similar: ${\$}$4933 and ${\$}$5148, respectively. In contrast, the mean costs for inpatient services for men and women (${\$}$4312 and ${\$}$3038, respectively) differed by over ${\$}$1200. Yet, similar to total treatment costs, sex differences in inpatient and outpatient treatment costs were not statistically significant.

## Discussion

In the US population, men and women have demonstrated different health care utilization and mortality associated with nutrition-related conditions including diabetes and CVD (8, 9). Diabetes elevates the risk of costly medical conditions, including CVD, stroke, and kidney disease (22). Women who are diagnosed with diabetes have a higher morbidity rate (in terms of urinary tract/kidney infections and abnormal lipids) than men who are diagnosed with diabetes (15). Men and women seem to have similar rates of CVD mortality due to diabetes, when the mortality rates are adjusted for major CVD risk factors; however, CVD develops 7–10 y later in women than in men (23). Although CVD is a major cause of death among men and women, it is underrecognized and undertreated among women (23).

AI/ANs are more likely to experience diabetes than are members of the general population (2). Compared with the US population with diabetes, AI/AN persons with diabetes experience a higher prevalence of comorbidities, particularly renal failure, neuropathy, and lower-extremity amputation (1). What is known about sex differences in diabetes and CVD is from the SHS—a study of AIs aged 45–74 y who lived in 13 communities in Arizona, Oklahoma, South Dakota, and North Dakota (13, 24). It has found that overall AI men have higher CVD mortality and morbidity and that AI women with diabetes incur a higher CVD risk than AI men (13, 24). The current study expands our understanding of sex differences between younger and older adult AI/AN persons’ health status; it also examines sex differences by health service use and costs.

The health status trends that we found in the Northern Plains IHS Service Unit are consistent with the SHS in that men have a higher prevalence of CVD (24). We also found that men had higher prevalence of substance use disorders than women, and women had higher levels of non–substance use mental health disorders, which may indicate gendered expression of comorbid mental health. The sociological literature suggests that men and women in the US general population express mental health through different routes; women are more likely to internalize stress and express their problems through depression and anxiety, whereas men are more likely to express their stress through behaviors like substance abuse (25, 26). Future research should investigate the role of gendered coping and lifestyle risk factors among AI/AN persons.

Differences in utilization and treatment costs between AI men and women with diabetes did not reach statistical significance; the lack of significance may be due in part to the sample size. Although sex differences in treatment costs were nonsignificant, total treatment costs for males were approximately ${\$}$900 higher than costs for females. These differences were primarily due to higher costs among males for inpatient utilization. Interestingly, despite having higher levels of cardiovascular conditions, men did not have higher use of primary, urgent, or emergency services. These differences merit future study.

The similar prevalence of diabetes by sex in this population may be influenced by the presence of a Special Diabetes Program for Indians (SDPI) that provides resources for preventive services and early detection (27). SDPI programs implement diabetes-related activities and services based on local needs and priorities, including ongoing opportunities for diabetes screening at community events (28). SDPI programs have seen hemoglobin A1c and cholesterol significantly improve among AI/ANs diagnosed with diabetes, whereas the Alaska Native Medical Center saw improvement in blood pressure (29–31). Specifically, SDPI program sites have improved team-based diabetes clinical care which has supported comorbidity reduction (30). Along with the increase of diabetes clinical teams, SDPI programs have also increased access to nutrition services, registered dietitians, and physical activity specialists for adults. One strength of a team-based patient care approach that is coupled with population management strategies is that it can connect individual patients to community food resources (32).

In addition, across AI/AN communities in the United States there are a growing number of programs that aim to improve AI community food environments to prevent diet-related conditions for future generations. For example, the THRIVE study, funded by the National Heart, Lung, and Blood Institute, supported Oklahoma AI communities in improving access to healthy foods in Tribally owned convenience stores, resulting in increased healthy food purchases among AIs in these communities (33). There are also programs to promote food sovereignty, often defined as a community's right to control their own food system, and developed to connect individual Tribal members to traditional and locally sourced fruit and vegetables (33, 34). These programs take a broad approach to improving nutrition that encompasses policy, systems, and environmental strategies (35) and show great promise in closing the gap on the significant diet-related disparities AI communities experience.

### Limitations

This study has several limitations. We highlight 2 main ones. Our analysis was limited by the size of the study population, which reduced our statistical power, and the findings are only generalizable to the active users of the Northern Plains IHS Service Unit. Health care utilization and treatment cost estimates are for IHS services and services paid for by IHS through PRC; they exclude utilization and costs for services obtained at non-IHS providers and not paid for by PRC. Nevertheless, the treatment cost estimates reflect the costs of providing services within the IHS service system. Given the limited knowledge of sex differences in diabetes prevalence, and of comorbidities, health care utilization, and treatment costs among AI/ANs, our study contributes to this understanding.

## Conclusions

Our study examined data from a Northern Plains IHS Service Unit to understand sex differences in diabetes prevalence, comorbidities, health care utilization, and treatment costs. Unlike the national trends where men have a slightly higher prevalence of diabetes than women, we found similar prevalence of diabetes by age and sex (2). However, the prevalence of some comorbidities varied by sex; AI men had a statistically higher prevalence of hypertension, CVD, and substance use disorders than women, and a lower prevalence of non–substance use mental health disorders. Among ages 55 y and older, we saw similar prevalence trends. These data are important to foreground AI health disparities and effectively plan prevention and treatment efforts.

## Notes

Supported by Patient-Centered Outcomes Research Institute contract AD-1304-6451 (to JOC), Agency for Healthcare Research and Quality contract 290-2006-00020-I (to JOC, Research PI), and Indian Health Service grant U26IHS300285/01 (to JOC).

Author disclosures: KRH, JR, VBBJ, TT, CW, SMM, and JOC, no conflicts of interest.

This article appears as part of the supplement “Proceedings of the Third Annual Conference on Native American Nutrition,” sponsored by the Shakopee Mdewakanton Sioux Community’s Seeds of Native Health campaign through a gift to the University of Minnesota. The guest editors of the supplement, Treena Delormier and Mindy S Kurzer, have no conflicts of interest. The opinions expressed in this publication are those of the authors and are not attributable to the sponsors or the publisher, Editor, or Editorial Board of *Current Developments in Nutrition*. Publication costs for this supplement were defrayed in part by the payment of page charges.

Abbreviations used: AI, American Indian; AN, Alaska Native; Cost Report, Centers for Medicare & Medicaid Services Cost Report; CVD, cardiovascular disease; ECP, education, case management, and advanced practice pharmacy services; FY2010, fiscal year 2010; IHS, Indian Health Service; IRB, institutional review board; NPIRS, National Patient Information Reporting System; PRC, Purchased and Referred Care; SHS, Strong Heart Study; SDPI, Special Diabetes Program for Indians.
